# PFresGO: an attention mechanism-based deep-learning approach for protein annotation by integrating gene ontology inter-relationships

**DOI:** 10.1093/bioinformatics/btad094

**Published:** 2023-02-16

**Authors:** Tong Pan, Chen Li, Yue Bi, Zhikang Wang, Robin B Gasser, Anthony W Purcell, Tatsuya Akutsu, Geoffrey I Webb, Seiya Imoto, Jiangning Song

**Affiliations:** Department of Biochemistry and Molecular Biology, Biomedicine Discovery Institute, Monash University, Melbourne, VIC 3800, Australia; Department of Biochemistry and Molecular Biology, Biomedicine Discovery Institute, Monash University, Melbourne, VIC 3800, Australia; Department of Biochemistry and Molecular Biology, Biomedicine Discovery Institute, Monash University, Melbourne, VIC 3800, Australia; Department of Biochemistry and Molecular Biology, Biomedicine Discovery Institute, Monash University, Melbourne, VIC 3800, Australia; Department of Veterinary Biosciences, Melbourne Veterinary School, The University of Melbourne, Parkville, VIC 3010, Australia; Department of Biochemistry and Molecular Biology, Biomedicine Discovery Institute, Monash University, Melbourne, VIC 3800, Australia; Bioinformatics Center, Institute for Chemical Research, Kyoto University, Uji 611-0011, Japan; Monash Data Futures Institute, Monash University, Melbourne, VIC 3800, Australia; Division of Health Medical Intelligence, Human Genome Center, Institute of Medical Science, The University of Tokyo, Minato-ku, Tokyo 108-8639, Japan; Collaborative Research Institute for Innovative Microbiology, The University of Tokyo, Bunkyo-ku, Tokyo 113-8657, Japan; Department of Biochemistry and Molecular Biology, Biomedicine Discovery Institute, Monash University, Melbourne, VIC 3800, Australia; Bioinformatics Center, Institute for Chemical Research, Kyoto University, Uji 611-0011, Japan; Monash Data Futures Institute, Monash University, Melbourne, VIC 3800, Australia

## Abstract

**Motivation:**

The rapid accumulation of high-throughput sequence data demands the development of effective and efficient data-driven computational methods to functionally annotate proteins. However, most current approaches used for functional annotation simply focus on the use of protein-level information but ignore inter-relationships among annotations.

**Results:**

Here, we established PFresGO, an attention-based deep-learning approach that incorporates hierarchical structures in Gene Ontology (GO) graphs and advances in natural language processing algorithms for the functional annotation of proteins. PFresGO employs a self-attention operation to capture the inter-relationships of GO terms, updates its embedding accordingly and uses a cross-attention operation to project protein representations and GO embedding into a common latent space to identify global protein sequence patterns and local functional residues. We demonstrate that PFresGO consistently achieves superior performance across GO categories when compared with ‘state-of-the-art’ methods. Importantly, we show that PFresGO can identify functionally important residues in protein sequences by assessing the distribution of attention weightings. PFresGO should serve as an effective tool for the accurate functional annotation of proteins and functional domains within proteins.

**Availability and implementation:**

PFresGO is available for academic purposes at https://github.com/BioColLab/PFresGO.

**Supplementary information:**

Supplementary data are available at *Bioinformatics* online.

## 1 Introduction

Proteins are indispensable macromolecules that play fundamental roles in many activities and biological functions in living cells, such as maintaining normal metabolism, transporting nutrients, transducing signals and catalyzing biochemistry interactions (Lee *et al.*, 2007). To infer general or specific functions of proteins and to establish their relationships, standardized classification schemes (Ouzounis *et al.*, 2003), such as the enzyme classification (EC) System (Bairoch, 2000), Kyoto Encyclopedia of Genes and Genomes (KEGG) (Kanehisa *et al.*, 2021) and Gene Ontology (GO) (The Gene Ontology Consortium, 2008) have been developed. To date, GO is a widely accepted and used system for the functional annotation of proteins (i.e. gene products). GO terms are organized hierarchically in a directed acyclic graph (DAG), according to protein relationships and are divided into three non-overlapping branches, namely molecular function (MF), biological process (BP) and cellular component (CC).

The rapid accumulation of protein datasets through the use of genomic, transcriptomic and proteomic techniques has resulted in an exponential growth in demand for high-throughput and reliable functional annotation of such datasets (Hasin *et al.*, 2017). For example, the UniProt database (The UniProt Consortium, 2021) contains more than 200 million protein sequences, but <1% of these entries have been fully annotated (Gligorijevic *et al.*, 2021), which relates to major limitations (in terms of throughput, time and cost) associated with the conventional approach of annotating proteins using laboratory-based methods and information from published literature. To circumvent these constraints, computational methods have been established to predict the functions of proteins represented in large datasets (Sharma *et al.*, 2022). These include homology-based, machine-learning-based and deep-learning-based methods. Homology-based methods rely on the comparison of protein sequences using, for example, BLAST (Ye *et al.*, 2006), because evolutionarily related proteins tend to have similar functions, although minor mutations can significantly alter protein structure and function.

Compared with homology-based methods, conventional machine-learning approaches, such as support vector machines (Cai *et al.*, 2003) and random forest (Chen and Ishwaran, 2012), and deep-learning-based approaches (Sapoval *et al.*, 2022) are reported to exhibit a superior prediction performance. Most deep-learning methods treat the annotation of protein function as a multi-label prediction task, where protein information is used as the model input and the predicted GO terms represent outputs, disregarding the correlations of GO labels. Although GO terms and their hierarchical structure have been measured based on semantic similarity (Edera *et al.*, 2022) and applied in various studies, there are limited studies that explicitly account for the GO term inter-relationships. DeepGO (Kulmanov *et al.*, 2018) constructed a deep-learning classification model that resembled the structure and dependencies between GO classes to refine features on each distinction present in the GO. Another tool, DEEPred (Sureyya Rifaioglu *et al.*, 2019), applied a stack of multi-task feed-forward networks according to the inheritance relationships of the GO system for protein function prediction. DeeProtGO (Merino *et al.*, 2022), which is a feed-forward deep neural network for predicting GO terms, integrated the GO knowledge represented by means of normalized co-occurrence vectors. Meanwhile, DeepGOZero (Kulmanov and Hoehndorf, 2022) combined a model-theoretic approach for learning ontology embedding, using the axioms of the GO to constrain function prediction. TALE (Cao and Shen, 2021) employed a transformer-based deep-learning model with a joint embedding of sequence inputs and hierarchical function labels. While it remains a great challenge regarding how to effectively capture the GO term inter-relationships, a recent study (Duong *et al.*, 2020) shows that incorporating the hierarchical structure of GO graphs can enable the annotation model to emphasize on the GO label distribution, thereby benefitting the final prediction.

In this article, we propose a novel, attention-based approach, termed PFresGO, for protein function annotation by leveraging both protein residual-level representations and GO architecture. PFresGO uses sequence information of the query proteins as input; it takes the protein sequence embedding encoded by the pre-trained language model as well as GO terms embedding as inputs and delivers a probability of protein function by calculating the correlation between protein features and individual GO terms via an attention mechanism. Our findings show that PFresGO performs better than existing methods across all GO categories and benefits markedly from the incorporation of GO hierarchical structure information. The interpretation of annotation results is enhanced through the location of functionally relevant residues/domains in protein sequences via the analysis of attention weights.

## 2 Materials and methods

### 2.1 Functional annotation data and GO graphs

We employed the curated dataset from the study of DeepFRI—a graph convolutional network (Gligorijevic *et al.*, 2021). This dataset of 36 641 protein sequences provided the coverage of 2752 GO terms across MF (*n* = 489 terms), BP (1943) and CC (320), with each GO term linked to >50 non-redundant Protein Data Bank (PDB) chains. This dataset was further divided into training (∼80%; 29 902 sequences), validation (∼10%; 3323 sequences) and test (∼10%; 3416 sequences) datasets for the training, optimization and evaluation of the model, respectively. For the test dataset, only proteins with at least one trusted functional annotation in each of the three GO categories was selected. All annotations represented in the test dataset were experimentally validated, and the maximum length of protein chains was limited to 1000. CD-HIT (Fu *et al.*, 2012) was applied to ensure that there were no redundant PDB chains between training and test datasets using varying sequence identity thresholds. The relationship among GO terms was illustrated as a DAG. A filtered version (based on the dataset) of the GO.obo format file describing the hierarchical related structure of GO terms was downloaded from the GO resource website (http://geneontology.org/; data version: 1 June 2020) (Day-Richter *et al.*, 2007), which ensures consistency in annotation with previous works for impartial comparisons.

### 2.2 Input features

#### 2.2.1 Protein sequence embedding

Given a protein sequence *S* with *l* residues, we first used one-hot embedding to represent the protein sequence *S*. Specifically, each residue in the protein sequence was embedded into a 26-dimension vector (including the 20 standard amino acids, 5 non-standard amino acids and 1 padding symbol). The one-hot encoding procedure was followed by a fully connected layer with hidden dimension $d_{0}$ to generate an embedding matrix $E_{1}\in R^{l\times d_{0}}$. We also utilized a deep-learning language model ProtT5 (Elnaggar *et al.*, 2021), which had been pre-trained on datasets comprising 393 billion amino acids, to encode the protein sequence *S* with *l* residues into the residue-level protein sequence feature embedding $E_{2}\in R^{l\times d_{1}}$, where $d_{1}$ is 1024 by default. The encoded residue-level feature vector comprises the information of individual residue and its immediate context, and constraints of protein global structure and protein function.

#### 2.2.2 GO term embedding

Gene ontology (GO) is a commonly used classification scheme in terms of annotating protein functions. Here, we applied the pre-trained model Anc2vec (Edera *et al.*, 2022) to generate the compact GO term embedding as the initial input of PFresGO. Anc2vec is a neural network-based protocol that considers the preservation of ontological uniqueness, ancestors’ hierarchy and sub-ontology membership to embed GO terms. More specifically, each GO term $G_{i}$ is embedded into a $d_{0}$ dimensional label representation vector, where $d_{0}$ is the predefined hidden dimension.

### 2.3 The autoencoder module

The autoencoder module (Ng, 2011) was used to reduce the high-dimension residue-level protein data to feature vectors of hidden dimension $d_{0}$. The module comprises two submodules, including an encoder submodule and a decoder submodule; each is composed of two layers of neurons. The encoder submodule transfers the high-dimension input data into a low-dimensional latent space, while the decoder submodule converts the low-dimensional vector back to the original space, reversely. The dimension-reduced feature vector in the latent space is represented as compressed low-dimension embedding of the original input.

The output of the encoder submodule can be computed using

(1) $$Z_{\mathrm{en}}^{i+1}=\mathrm{ReLU}\left(W_{\mathrm{en}}^{i+1}\times Z_{\mathrm{en}}^{i}+b_{\mathrm{en}}^{i+1}\right),$$

where $W_{\mathrm{en}}^{i+1}$ and $b_{\mathrm{en}}^{i+1}\;$denote the learned weights and the bias of i+1 th encoder layer. The rectified linear unit (ReLU) is a non-linear activate function ReLU=max⁡(x,0). The output of the previous encoder layer $Z_{\mathrm{en}}^{i}$ serves as the input of the following i+1 th encoder layer. Specifically, the initial input to the encoder submodule is the residue-level protein sequence features, i.e. $Z_{\mathrm{en}}^{0}=E_{2}\in R^{l\times d_{1}}$, where $d_{1}=1024$. The number of neurons in the second encoder layer is the predefined hidden dimension $d_{0}$.

The decoder submodule takes the reduced dimension embedding $Z_{\mathrm{en}}^{2}$ as input and aims to recover the embedded feature vector into the original dimension. The output of the decoder submodule can be computed as:

(2) $$Z_{\mathrm{de}}^{i+1}=\mathrm{ReLU}\left(W_{\mathrm{de}}^{i+1}\times Z_{\mathrm{de}}^{i}+b_{\mathrm{de}}^{i+1}\right),$$

where $W_{\mathrm{de}}^{i+1}$ and $b_{\mathrm{de}}^{i+1}\;$represent the learned weights and the bias of i+1th decoder layer, respectively. The decoder module consists of two neural network layers. The output of the previous decoder layer $Z_{\mathrm{de}}^{1}$ is used as the input of the following decoder layer. The final optimization goal of the autoencoder is to minimize the reconstruction error (squared error) between the initial encoder input and the reconstructed decoder output:

(3) $$\mathrm{loss}=\sum_{n}\sum_{d}{\left||Z_{\mathrm{en}}^{0}\left(n,d\right)-Z_{\mathrm{de}}^{2}(n,d)|\right|}^{2}.$$

Given the protein feature vector $E_{2}\in R^{l\times d}$, we computed the encoder submodule output $E_{2}\in R^{l\times d_{0}}$ as the compressed residue-level embedding, which is then added with $E_{1}\in R^{l\times d_{0}}$ for the final protein residue-level embedding $E\in R^{l\times d_{0}}$.

### 2.4 The multi-head attention module

The functional annotation of proteins is a multi-label classification task. The prediction algorithm should, therefore, consider the relationships among GO terms. Theoretically, proteins perform specific biological functions relying on spatially aggregated functional residues, such as ligand-binding sites of proteins and catalytic residues in enzymes (Lichtarge *et al.*, 1996). We then expect PFresGO to be able to dynamically focus on functional residues to capture the relationship among GO terms and key functional regions within protein sequences, thereby enabling the final predictions of protein functions. With this goal in mind, we integrated two multi-head attention operations to enable PFresGO to simultaneously capture relevant feature projections from multi-subspaces. The main principle of the multi-head attention mechanism is to calculate the scaled dot-product attention as follows:

(4) $$\mathrm{Attention}\left(Q,K,V\right)=\mathrm{softmax}\left(\frac{Q\times K^{T}}{\sqrt{d_{K}}}\right)V,$$

where *Q*, *K* and *V* refer to the query, key and value matrix transformed from the attention layer input, respectively, and $d_{K}\;$represents a constant of the key dimension as a scalar factor.

The first multi-head attention operation encourages the model to automatically capture the correlations between GO terms and then update the GO term embedding accordingly. Given an input of GO term embedding $G\in R^{m\times d_{0}}$, the updated GO term embedding $\bar{G}$ can be calculated as

(5) $${\mathrm{head}}_{i}=\mathrm{Attention}(GW_{i}^{Q},GW_{i}^{K},GW_{i}^{V}),$$

(6) $$G_{1}=\mathrm{Contact}\left({\mathrm{head}}_{1},\ldots ,{\mathrm{head}}_{n}\right)W^{O},$$

(7) $$\bar{G}=\mathrm{LN}\left(G_{1}+G\right),$$

where ${\mathrm{head}}_{i}$ indicates the *i*th attention head, with *n* heads in total. The learned weights $W_{i}^{Q}\in R^{d_{0}\times d_{k}}$, $W_{i}^{K}\in R^{d_{0}\times d_{k}}$ and $W_{i}^{V}\in R^{d_{0}\times d_{V}}$ are used to project the input GO term embedding $G\in R^{m*d_{0}}$ into the corresponding query matrix $Q\in R^{m\times d_{k}}$, the key matrix $K\in R^{m\times d_{k}}$, and the value matrix $V\in R^{m\times d_{V}}$, respectively. The *n* attention matrix computed based on *Q*, *K* and *V* are then concatenated and multiplied for the final output matrix $W_{i}^{O}\in R^{nd_{V}\times d_{0}}$, to obtain the updated GO term embedding $G_{1}\in R^{m\times d_{0}}$. Then, a residual connection as well as a layer normalization procedure, was applied to obtain $\bar{G}\in R^{m\times d_{0}}$.

Another multi-head attention mechanism was applied, where the model takes GO terms as a query to detect specific protein features important for protein function annotation. The protein embedding is zero-padded if the protein chain consists of <1000 residues. Given a zero-padded protein feature embedding $\bar{E}\in R^{L\times d_{0}}\;(L=1000)$, we first calculated the attention between the protein feature and GO labels:.

(8) $${\mathrm{head}}_{i}=\mathrm{Attention}(\bar{G}W_{i}^{Q},\bar{G}W_{i}^{K},\bar{G}W_{i}^{V}),$$

(9) $$G_{2}=\mathrm{Contact}\left({\mathrm{head}}_{1},\ldots ,{\mathrm{head}}_{n}\right)W^{O},$$

(10) $$\hat{G}=\mathrm{LN}\left(G_{2}+\bar{G}\right),$$

where ${\mathrm{head}}_{i}$ indicates the *i*th attention head, with *n* heads in total. Similarly, the learned weights $W_{i}^{Q}\in R^{d_{0}\times d_{k}}$, $W_{i}^{K}\in R^{d_{0}\times d_{k}}$ and $W_{i}^{V}\in R^{d_{0}\times d_{V}}$ are used to project the input GO term embedding $\bar{G}\in R^{m\times d_{0}}$ into the corresponding query matrix $Q\in R^{m\times d_{k}}$, and project the residue-level protein feature embedding $\bar{E}\in R^{L\times d_{0}}$ into the key matrix $K\in R^{L\times d_{k}}$, and the value matrix $V\in R^{L\times d_{V}}$, respectively. The *n* attention matrix computed based on *Q*, *K* and *V* were then concatenated and multiplied for the final output matrix $W^{O}\in R^{nd_{V}\times d_{0}}$, to obtain the updated GO term embedding $G_{2}\in R^{m\times d_{0}}$. Again, a residual connection and layer normalization were applied to acquire $\hat{G}\in R^{m\times d_{0}}$. A feed-forward layer is followed to take the $d_{0}$ dimensional embedding $\hat{G}$ as input and perform two point-wise dense layers to obtain $\mathrm{FF}(\hat{G})\in R^{m\times d}$:

(11) $$\mathrm{FF}\left(\hat{G}\right)=\mathrm{ReLU}\left(\hat{G}W_{1}+b_{1}\right)W_{2}+b_{2},$$

where $W_{1}$, $W_{2}$, $b_{1}$ and $b_{2}$ are learnable weights and biases of two dense layers, respectively. Here, we linked two multi-head attention modules for MF and CC protein function annotation. For BP term, only one multi-head attention module is applied considering the memory limitation.

### 2.5 The GO term prediction module

This module computes the probability of each GO term. It formulates the multi-label task of protein function annotation as a binary classification task. Specifically, it projects the individual GO term embedding feature into a probability value. In the first step, we performed a global pooling on the resulted vector $\mathrm{FF}\left(\hat{G}\right)$ by summing over the last dimension:

(12) $$h^{\mathrm{pool}}=\sum_{i=1}^{\hat{d}}\mathrm{FF}(\hat{G}).$$

We then computed the final probability distribution utilizing a fully connected layer with the *sigmoid* activation function from this pooled representation. The *m*-dimension output vector stands for the predicted probability of *m* GO terms:

(13) $$Y=\mathrm{sigmoid}(W\times h^{\mathrm{pool}}+b).$$

Given the true protein function GO label and the predicted probabilities, we minimized the binary cross-entropy loss to optimize the above process:

(14) $$L=-\frac{1}{N}\sum_{i=1}^{N}\sum_{j=1}^{\left|\mathrm{GO}\right|}y_{ij}\log\left({\hat{y}}_{ij}\right),$$

where *N* represents the total number of protein chains, |GO| is the total number of GO terms, $y_{ij}$ and ${\hat{y}}_{ij}$ represents the true value and the predicted probability of GO term *j* for protein chain *i*.

## 3 Results

### 3.1 PFresGO annotates protein function using GO term inter-relationships

**Fig. 1.**
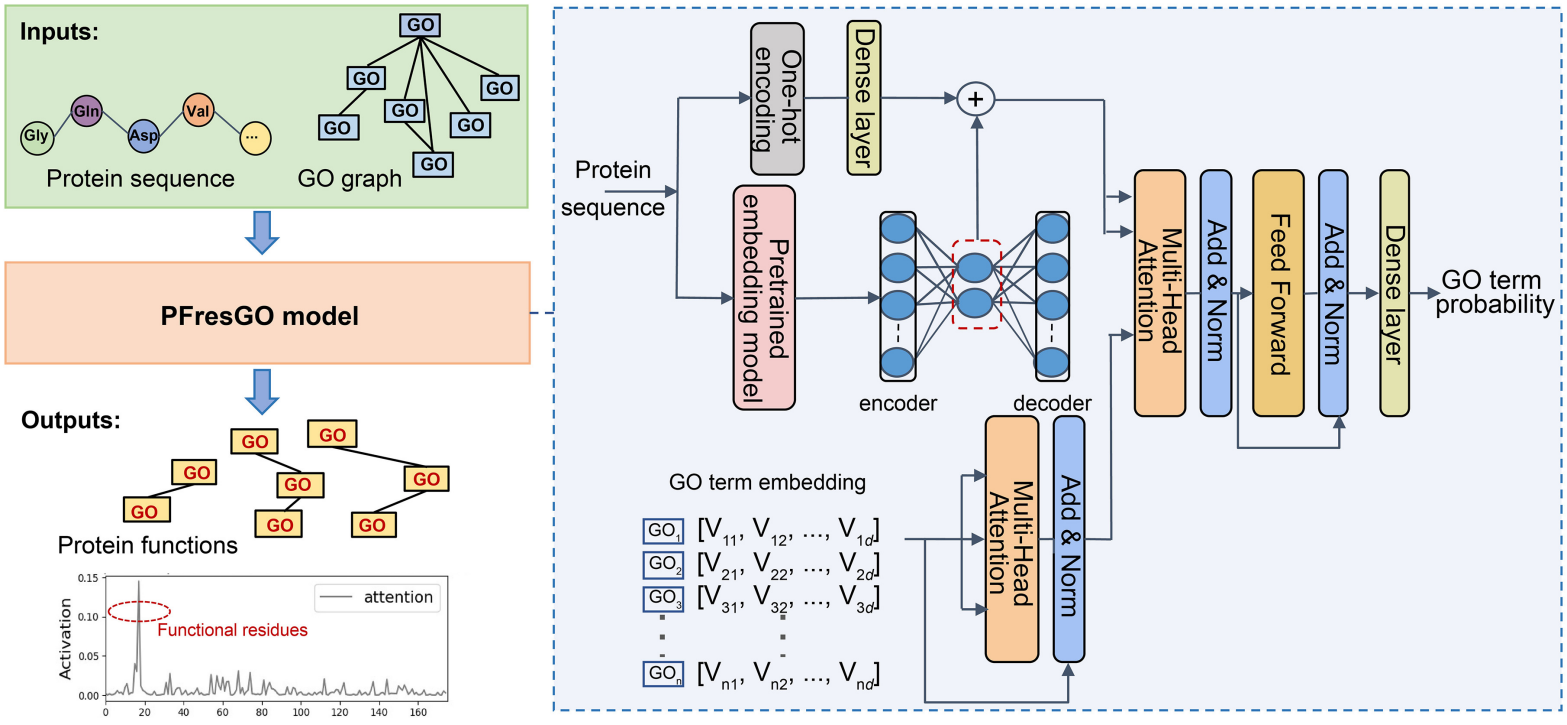
The architecture of PFresGO. The pre-trained language model (Elnaggar *et al.*, 2021) encodes amino acid sequences into protein feature embedding. An autoencoder module reduces the protein embedding to the hidden dimension d0, which adds with the projected one-hot sequence embedding to produce the residue-level protein representation. A self-attention operation is utilized to explore the relationships between GO term representations generated by Anc2vec (Edera *et al.*, 2022), a deep neural network for constructing GO term embedding and further updates the representations accordingly. A cross-attention operation is used to detect the correlation between the protein features and protein functions by taking gene ontology embedding as a query to detect related protein information, which is followed by linear layers to output the final GO term probability

First, we describe how PFresGO performs function annotations for a query protein. In brief, PFresGO contains three critical mechanisms to facilitate protein function prediction using GO terms, including a pre-trained protein language model, a GO inter-relationship self-attention model and a multi-head cross-attention mechanism. The architecture of PFresGO is illustrated in Figure 1. We utilized a pre-trained protein language model, ProtT5 (Elnaggar *et al.*, 2021), to encode informative protein sequence embedding, which is a novel natural-language-based model trained on >390 billion amino acids. We learned a compact representation for embedded protein vectors using an autoencoder to reduce these vectors to a hidden dimension, which was then added with the projected one-hot embedding of protein sequences as the final protein feature representation at the amino acid residue level.

To learn the inter-relationships of GO terms, we initially applied the deep-learning-based Anc2vec algorithm (Edera *et al.*, 2022) to generate a compact GO term embedding, according to the hierarchical structure of the GO graph. A multi-head self-attention operation was then used to capture the inherent semantic relations of GO terms automatically and to update the GO term embedding accordingly. An ‘Add and Norm’ operation was then conducted to facilitate and stabilize the algorithm training process. We then applied a multi-head cross-attention operation to project residue-level protein representations and GO embedding into a common latent space, where GO terms act as a query to detect the global protein sequence patterns as well as local functional residues. The resultant vectors were processed by the ‘Add and Norm’ operation and then fed into a feed-forward module constituting two fully connected layers. The final dense layer, in which the number of neurons equals the number of GO term labels, serves as the output layer and computes the probability of each protein function term. A detailed description of PFresGO implementation and optimization is provided in Supplementary Material.

### 3.2 PFresGO outperforms existing methods across all GO categories

We compared PFresGO to six previously proposed approaches: one sequence identity-based search method BLAST (Ye *et al.*, 2006), one protein domain-based function transfer approach FunFams (Das *et al.*, 2015) and four state-of-the-art deep-learning-based approaches DeepGO (Kulmanov *et al.*, 2018), DeepFRI (Gligorijevic *et al.*, 2021), TALE+ (Cao and Shen, 2021) and DeepGOZero (Kulmanov and Hoehndorf, 2022). Of these, BLAST has been extensively applied as the ‘standard’ sequence-based method in many studies. In addition, DeepFRI was applied for structure-based comparison of the text dataset. Supplementary Sections S2 and S4 describe details regarding performance measures and the comparison of distinct approaches.

The performance comparison results are provided in Table 1. Compared with the other methods assessed, PFresGO achieved a remarkable performance with $F_{\max}$ values of 0.6917, 0.5678 and 0.6737 for MF, BP and CC, respectively, while DeepGOZero (i.e. 0.7191) outperformed the other methods in terms of $F_{\max}$ for MF. In relation to AUPRC, PFresGO performed favorably compared with the other methods, achieving values of 0.6017, 0.2934 and 0.3612 for MF, BP and CC, compared with 0.1357, 0.0674 and 0.0973 for BLAST, respectively. PFresGO consistently outperformed other methods in terms of AUROC for MF and CC, and achieved a comparable AUROC value (0.8394) to that of DeepFRI (i.e. 0.8578) for BP. As for $S_{\min}$, FunFams outperformed the competing methods for BP and CC, while PFresGO was the top three best predictor for MF and CC. Taken together, the performance values in Table 1 show the effectiveness of the proposed deep-learning strategy in PFresGO for the annotation of protein function.

**Table 1.** Performance comparison of PFresGO and state-of-the-art methods for protein function prediction on the independent test dataset

Method	GO category	$F_{\max}$	AUPRC	AUROC	$S_{\min}$
BLAST	MF	0.3282	0.1357	0.7114	5.5119
	BP	0.3358	0.0674	0.6450	49.0130
	CC	0.4478	0.0973	0.6650	6.4769
DeepGO	MF	0.5772	0.3911	0.8599	4.1592
	BP	0.4934	0.1821	0.8080	44.4762
	CC	0.5941	0.2627	0.8617	5.6486
FunFams	MF	0.5721	0.3671	0.7506	3.8517
	BP	0.4997	0.2600	0.7091	**38.9009**
	CC	0.6265	0.2882	0.7677	**4.7833**
DeepFRI	MF	0.6246	0.4949	0.9147	3.7344
	BP	0.5402	0.2612	**0**.**8578**	41.9820
	CC	0.6126	0.2744	0.8837	5.4917
TALE+	MF	0.6624	0.5642	0.8844	3.2205
	BP	0.5539	**0.3021**	0.8105	39.9229
	CC	0.6099	0.3251	0.8486	5.3235
DeepGOZero	MF	**0.7191**	**0.6144**	0.8925	**3.0187**
	BP	0.5645	0.2944	0.7682	40.9241
	CC	0.5341	0.3146	0.7381	5.4340
PFresGO	MF	0.6917	0.6017	**0**.**9247**	3.5600
	BP	**0.5678**	0.2934	0.8394	41.3265
	CC	**0**.**6737**	**0.3612**	**0**.**8841**	5.1916

*Note*: The best performance on MF, BP and CC categories has been bolded.

### 3.3 Incorporating GO term inter-relationships improves functional annotation

The inter-relationships of GO terms are incorporated into PFresGO via two attention-based operations: the first operation automatically captures hierarchical information about GO graphs and updates the embedding accordingly; the second operation takes the embedding of each GO term as a query to explore potentially important protein features in the same latent space for the prediction of individual protein function terms.

**Fig. 2.**
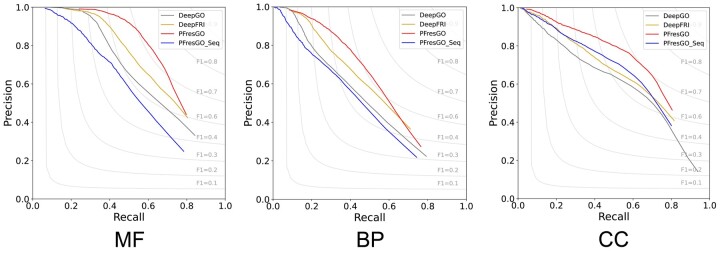
Precision-recall curves of methods DeepGO, DeepFRI, PFresGO and PFresGO_Seq on MF, BP and CC terms

To thoroughly delineate the effectiveness of incorporating GO terms inter-relationships, we built PFresGO_Seq by only feeding the extracted protein feature representation into a dense output layer. We compared the AUPRC values of PFresGO_Seq and PFresGO, as well as the other two baseline deep-learning methods—DeepGO and DeepFRI—across all GO categories on the test dataset (Fig. 2). Although PFresGO_Seq had a comparable performance to DeepGO for BP and to DeepFRI for CC ontology, PFresGO significantly outperformed all other methods assessed for all three GO categories (MF, BP and CC). These results show that PFresGO largely benefits from the strategy of incorporating GO term inter-relationships to functionally annotate proteins.

### 3.4 PFresGO shows superior performance in annotating protein function with different sequence identities and GO specificities

Here, we initially evaluated the ability of PFresGO to predictive using protein sequences with different identities, especially in the case of novel protein sequences with low sequence identities compared to the training dataset. We split the test dataset into five different groups with varying sequence identity thresholds: 30%, 40%, 50%, 70% and 95%, which are the maximum identity values of test sequences compared to the training dataset. We compared PFresGO with other models, including BLAST, DeepGO, FunFams, TALE+ and DeepFRI, using $F_{\max}$ and AUPRC on the same test datasets, split by sequence identity thresholds.

**Fig. 3.**
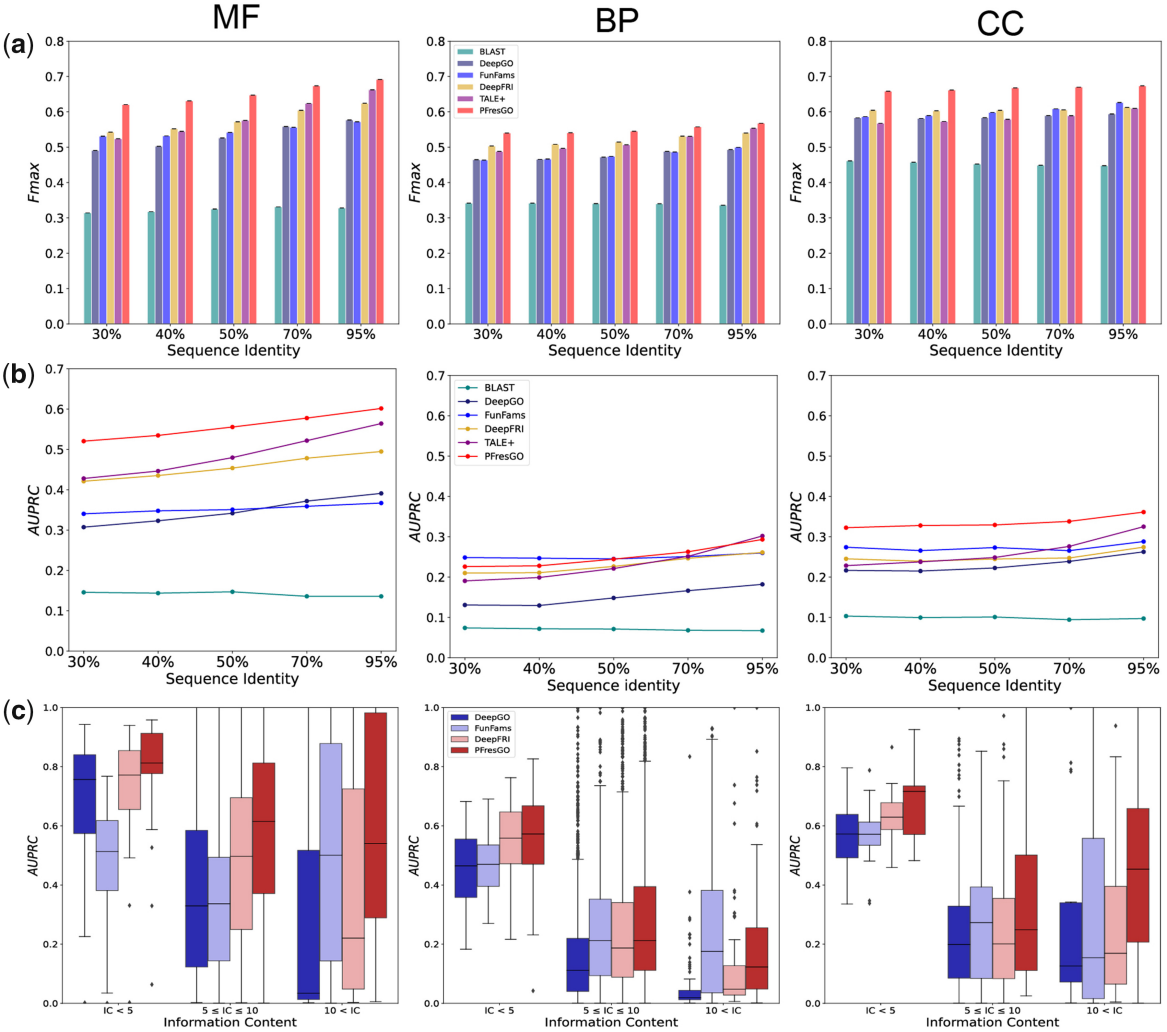
Performance comparison of (**a**) *F*_max_, (**b**) AUPRC on varying sequence identity and (**c**) distributions of AUPRC scores on varying IC across MF, BP and CC categories among different methods: Blast, DeepGO, FunFams, DeepFRI, TALE+ and PFresGO

As shown in Figure 3a and b, the $F_{\max}$ values of all methods improved with increased sequence identity for all three GO categories, while PFresGO consistently outperformed other methods, regardless of the sequence identity. PFresGO also has higher AUPRC values for both MF and CC for all sequence identity thresholds, even when the test proteins shared ≤30% identity with the training dataset. Although FunFams achieved a higher AUPRC value for BP for proteins sharing <40% identity to the training dataset, PFresGO outperformed other methods, achieving a higher AUPRC score for sequence identities ranging from 50% to 95%.

Subsequently, we investigated the performance of PFresGO when annotating GO terms with a high specificity. Here, we evaluated the specificity of GO terms according to their Shannon Information Content (IC).

(15) $$\mathrm{IC}\left({\mathrm{GO}}_{i}\right)=-\log_{2}\mathrm{Prob}({\mathrm{GO}}_{i}).$$

A higher IC value of the GO term corresponds to a higher specificity (i.e. a rarer occurrence). We separately stratified the GO terms within the MF, BP and CC categories into three groups according to their IC values and then compared the performance of PFresGO with that of other methods using these GO terms with distinct IC values. We recorded the AUPRC values of different methods across all IC cutoffs (Fig. 3c). Although all methods consistently showed sound performance when predicting protein GO terms for lower IC values, the high AUPRC value of PFresGO provided evidence of a marked advantage of integrating GO term relationships in the training process. PFresGO outperformed other methods in terms of AUPRC for GO terms, with high specificity (i.e. IC >10) for MF and CC branches; its performance was comparable with FunFams for GO terms with high specificity (IC >10) on the BP branch. PFresGO showed strong scalability and generalizability for functional annotation of novel query proteins with a limited sequence identity to those in training sets, and usually for annotation of GO terms with high specificity.

Furthermore, we analyzed the performance of PFresGO in cases where the proteins in test set did not share any homologous domains with those in training set. More specifically, we applied the ECOD classifier (file: ‘ecod.latest.domains.txt’, version: ‘20221014’) (Schaeffer *et al.*, 2017) to rigorously divide the training and test sets to eliminate most if not all evolutionary relationships (i.e. the H level). Please refer to the Supplementary Section S9 for detailed results. It can be seen that the performance of PFresGO dropped across all GO branches, indicating the use of protein domains as analytic units could improve the protein function annotation.

### 3.5 PFresGO locates residues linked to protein function annotation

**Fig. 4.**
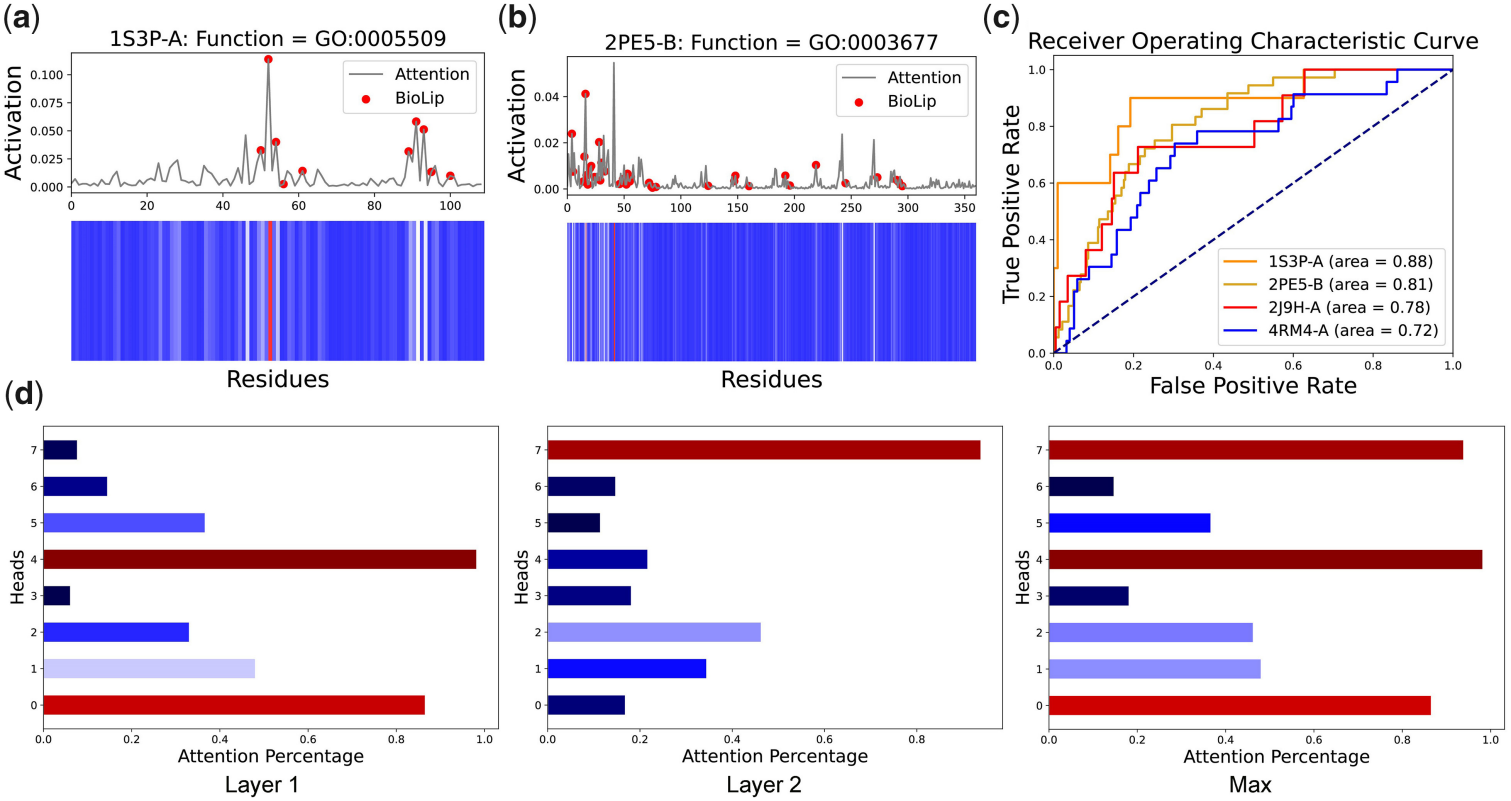
PFresGO locates functional residues based on attention weights: (**a**) attention weights of rat α-parvalbumin (PDB: 1S3P, Chain A) with function calcium ion binding (GO: 0005509), the dots correspond to calcium-binding residues annotated in BioLip; (**b**) attention weights of lactose operon repressor (PDB: 2PE5, Chain B) with function DNA binding (GO: 0003677); (**c**) ROC curves of residues identified by attention weights and functional residues of protein examples retrieved from BioLip; and (**d**) an example of the percentage of attention on binding sites. The left, medium and right bars show the percentage of attention of every head in attention Layer 1, Layer 2 and the maximum percentage of each head, respectively

As it has been reported that spatially aggregated functional residues play critical roles in protein functions (Lichtarge *et al.*, 1996), we assessed the ability of PFresGO to infer the location(s) of residues linked to protein function. Our hypothesis here is that PFresGO is capable of focusing more on protein residues that make more important contributions to protein function annotation with higher attention weights, and accordingly, such functionally important resides can be identified via the analysis of attention weights assigned by PFresGO. In Figure 4a, we illustrate an example of the visualization of averaged attention weights, which indicates that PFresGO correctly identified the sites in rat α-parvalbumin (PDB: 1S3P; Chain A) linked to ‘calcium ion binding’ (GO: 0005509). The grey line corresponds to the varying attention weights along the protein sequence, while the red dots represent the experimentally validated calcium-binding sites annotated in BioLip (Yang *et al.*, 2013). In Figure 4b, we provide another example where PFresGO correctly identified most sites of lactose operon repressor (PDB: 2PE5, Chain B) associated with DNA binding (GO: 0003677). We plotted the ROC curves for four examples of proteins with known functional residues to measure the consistency between important residues identified by PFresGO with the genuine protein functional residues annotated in BioLip in Figure 4c. Specifically, we calculated the attention weights of the proteins rat α-parvalbumin (PDB: 1S3P; Chain A), lactose operon repressor (PDB: 2PE5; Chain B), glutathione S-transferase (PDB: 2J9H; Chain A) and a putative cytochrome (PDB: 4RM4; Chain A) for the terms ‘calcium ion binding’ (GO: 0005509), ‘DNA binding’ (GO: 0003677), ‘glutathione transferase activity’ (GO: 0004364) and ‘heme binding’ (GO: 0020037), and compared them with the binary representation of function sites retrieved from BioLip. Despite the lack of functionally active sites or related information in the training process, the functional sites inferred by the attention weights and those within BioLip are highly correlated (Fig. 4c).

To explore how the attention weights of every head align with known protein functional residues, we defined the following function to compute the percentage of high-confidence attentions that are indicative of protein functional residues:

(16) $$P_{\alpha }\left(f\right)=\frac{\sum_{i=1}^{\left|X\right|}f(i)\times A_{\alpha_{i}>\theta }}{\sum_{i=1}^{\left|X\right|}A_{\alpha_{i}>\theta }},$$

where f(i) is an indicator function that returns 1 if the *i*th residue in the protein sequence *X* is annotated as a functional site in the BioLip database; otherwise returns 0, θ (θ=0) represents a threshold used for filtering out the high-confidence residues, and $A_{\alpha_{i}>\theta }$ indicates the attention weights of the high-confidence residues ($\alpha_{i}>\theta$).

Figure 4d shows the proportion of attention weights for protein rat α-parvalbumin for two attention layers of PFresGO. The first head in attention layer 1 almost paid all of its attention to the functional residues and ignored other general residues. Further, the fifth head in attention Layer 1 and the eighth head in attention Layer 2 paid > 80% of attention to functional residues. Please refer to the Supplementary Material for the attention percentage analysis on other cases. Considering that functional sites of protein are often evolutionarily conserved to sustain function across the tree-of-life, our analysis demonstrates that PFresGO can accurately infer protein function at a residue level.

## 4 Discussion and conclusion

In this study, we established PFresGO—an attention-based deep-learning method to tackle the multi-label protein function annotation challenge utilizing both the protein sequence information and hierarchical GO structures. PFresGO requires no information other than protein sequences for functional annotation, which is particularly convenient for newly identified proteins. Our evaluation of an independent test dataset showed that PFresGO achieved a superior prediction performance compared with current, ‘state-of-the-art’ sequence-based methods, and importantly, structure-guided approaches for all GO categories, indicating that the incorporation of hierarchical structures of GO graphs for the prediction of protein functions is effective. Importantly, PFresGO functionally annotates proteins with no requirement for multiple sequence alignment (MSA) (Edgar and Batzoglou, 2006). Although MSA has been routinely used to support protein structure and functional modeling, the inference of protein homology through sequence alignment alone is not feasible on a genome-wide scale. Circumventing the computational bottleneck imposed by MSA, PFresGO annotates proteins by identifying sequence patterns and functional residues, and the findings here show that PFresGO consistently achieves confident annotation results.

We demonstrated the effectiveness of integrating a pre-trained deep-learning language model and the hierarchical structure of GO terms for function annotation. On the other hand, there is a caveat when engaging the attention-based mechanism, the use of which can result in substantial memory consumption, which can further limit the number of GO terms that can be annotated; however, the annotation performance using GO terms of high specificity significantly benefits from the integration of structure information from GO graphs. Significantly, PFresGO can also infer functional sites in protein by assessing the attention weightings of individual amino acid residues. A case study showed that the distribution of attention weights along a protein sequence is readily interpretable in relation to functionally relevant amino acid residues or domains. We conducted a multiple attention analysis of the functions of select proteins and illustrated that important residues identified by PFresGO with high attention weights accord well experimental data in the BioLip database. Based on these findings, we expect that PFresGO will serve as a useful tool for the functional annotation of proteins and the identification of functional sites in proteins, which will be beneficial given the ever-expanding genomic, proteomic and transcriptomic datasets.

## Supplementary Material

btad094_Supplementary_Data

## Data Availability

All data underlying this work, including source code, is freely available at https://github.com/BioColLab/PFresGO.
