# Supplementary material for: PFresGO: an attention mechanism-based deep-learning approach for protein annotation by integrating gene ontology inter-relationships
Source: Bioinformatics. 2023 Feb 16;39(3):btad094. doi: 10.1093/bioinformatics/btad094 (PMC9978587; doi:10.1093/bioinformatics/btad094)
Supplement: btad094_Supplementary_Data [file btad094_supplementary_data.docx]

**PFresGO: an attention mechanism-based deep-learning approach to annotate protein function by integrating gene ontology inter-relationships**

Tong Pan^1^, Chen Li^1^, Yue Bi^1^, Zhikang Wang^1^, Robin B. Gasser^2^, Anthony W. Purcell^1^, Tatsuya Akutsu^3^, Geoffrey I. Webb^4,*^, Seiya Imoto^5,6,*^, and Jiangning Song^1,3,4,*^

^1^Biomedicine Discovery Institute and Department of Biochemistry and Molecular Biology, Monash University, Melbourne, VIC 3800, Australia; ^2^Department of Veterinary Biosciences, Melbourne Veterinary School, The University of Melbourne, Parkville, VIC 3010, Australia; ^3^Bioinformatics Center, Institute for Chemical Research, Kyoto University, Uji 611-0011, Japan; ^4^Monash Data Futures Institute, Monash University, VIC 3800, Australia; ^5^Division of Health Medical Intelligence, Human Genome Center, Institute of Medical Science, The University of Tokyo, Minato-ku, Tokyo, Japan; ^6^Collaborative Research Institute for Innovative Microbiology, The University of Tokyo, Bunkyo-ku, Tokyo, Japan.

*To whom correspondence should be addressed: [Jiangning.Song@monash.edu](mailto:Jiangning.Song@monash.edu), [Geoff.Webb@monash.edu](mailto:Geoff.Webb@monash.edu), [imoto@ims.u-tokyo.ac.jp](mailto:imoto@ims.u-tokyo.ac.jp).

**Supplementary materials**

1. PFresGO implementation

PFresGO was implemented using TensorFlow 2.4.1 (https://www.tensorflow.org/). We trained the PFresGO model on a single NVIDIA GeForce RTX 3090 GPU during the supervised training stage. A binary-cross entropy was used as a loss function to optimize the entire algorithm via the Adam optimizer with a learning rate of 1e-4 for 100 epochs. During the training process, sequences shorter than 1000 amino acids were padded zeros to reach the length of 1000. The number of multi-head attention modules is 2, 1, and 2 during training for MF (Molecular Function), BP (Biological Process), and CC (Cellular Component), respectively. We set the batch size as 32 and used a hidden dimension of 128, a feed-forward layer dimension of 1024, and a multi-head attention with 8 heads. An early stopping mechanism was applied with 5 patience of epochs in case of overfitting.

(1) The autoencoder architecture:

**Input**: protein embedding generated by pretrained model ProtT5: S_prott5 = (n_samples, L, 1024).

Encoder:

Dense (dim = 1024, use_bias = True)

Activation (ReLU)

Dense (dim = 256, use_bias = True)

Activation (ReLU)

Dense (dim = 128, use_bias = True)

Activation (ReLU)

Decoder:

Dense (dim = 128, use_bias = True)

Activation (ReLU)

Dense (dim = 256, use_bias = True)

Activation (ReLU)

Dense (dim = 1024, use_bias = True)

Activation (ReLU)

Optimization: loss = ’mse’; optimizer = adam (lr= 0.0001, β_1=0.95, β_2=0.95); batch_size = 256; epochs = 1000; early stopping (patience=5).

(2) The PFresGO architecture

**Input**: One-hot encoding of sequences S = (n_samples, L, 26); Sequence vector generated by Pre-trained ProtT5: S_prott5 = (n_samples, L, 1024); GO term embedding generated by Pre-trained Anc2vec: G = ( n_samples, m, 128).

Residue-level protein embedding:

S_emb1 = Dense (dim = 128, use_bias = True)(S)

Activation (ReLU)

S_emb2 = Encoder (S_prott5)

Add() (S_emb1, S_emb2)

Multi-head attention module:

Multi-Head Attention layer(d_model = 128, num_heads = 8)

Add & LayerNorm()

Multi-Head Attention layer(d_model = 128, num_heads = 8)

Add & LayerNorm()

Feed-forward layer(d_model = 128, dff = 1024)

Add & LayerNorm()

GO term prediction output module:

Residual Sumpooling()

Dense(num_class = m, use_bias = True)

Activation (sigmoid)

Optimization: loss = ’binary_crossentropy’; optimizer = adam (lr= 0.0001, β_1=0.95, β_2=0.95); batch_size = 32; epochs = 100; early stopping (patience=5).

2. Evaluation measures

Four measures, including $F_{max}$, AUPRC, AUROC and $S_{min}$ were used to evaluate the performance of PFresGO. All three measures have been widely used, especially in the CAFA (Critical Assessment of protein Function Annotation) challenge. $F_{max}$ ([0,1]) is a protein-centric evaluation measure, which is defined as the maximum f1 score over all proteins:

$F_{max}=\max_{t} (\frac{2Avgpr(t)\times Avgrc(t)}{Avgpr\left( t \right)+Avgrc(t)})$,

where *Avgpr(t)* and *Avgrc(t)* indicate averaged precision and recall with respect to specific threshold *t*.

AUPRC (area under the precision-recall curve) is commonly used to measure the precision and recall trade-off at each threshold, where precision and recall for each GO term can be computed as:

$pr_{f}(t)=\frac{\sum_{i} I(f\in P_{i}(t)\cap f\in T_{i})}{\sum_{i} I(f\in P_{i}(t))}$,

$rc_{f}(t)=\frac{\sum_{i} I(f\in P_{i}(t)\cap f\in T_{i})}{\sum_{i} I(f\in T_{i})}$.

We also used AUROC (area under the receiver operating characteristic curve). However, one should bear in mind that for unbalanced datasets like the protein function annotation task, AUPRC is commonly regarded as a more suitable evaluation measure than AUROC.

$S_{min}$ computes the semantic distance between real and predicted annotations based on information content of the classes. The information content IC(c) is computed based on the annotation probability of the class c:

$\mathrm{IC}\left( c \right)=-log(Pr(c|P(c)))$,

where P(c) is a set of parent classes of the class c. The $S_{min}$ is computed using the following formulas:

$S_{min}=\min_{t} \sqrt{{ru\left( t \right)}^{2}+{mi(t)}^{2}}$,

where ru(t) is the average remaining uncertainty and mi(t) is average misinformation:

$ru\left( t \right)=\frac{1}{n}\sum_{i=1}^{n} \sum_{C\in T_{i}-P_{i}(t)} IC(c)$,

$mi\left( t \right)=\frac{1}{n}\sum_{i=1}^{n} \sum_{C\in P_{i}(t)-T_{i}} IC(c)$.

3. A statistical summary of the training and test datasets

**Supplementary Table 1.** Statistical summary of the training and test datasets curated in this study.

| Data | MF | BP | CC |
| --- | --- | --- | --- |
| Train | 29902 | 29902 | 29902 |
| Validation | 3323 | 3323 | 3323 |
| Test | 3416 | 3416 | 3416 |
| GO terms | 489 | 1943 | 320 |

4. A brief introduction to the compared algorithms

**BLAST**: The Basic Local Alignment Search Tool (BLAST) (Ye, McGinnis and Madden, 2006) is a baseline widely accepted in CAFA. BLAST can be used to identify the protein sequence or region similarity. For protein function prediction, BLAST compares the sequence similarity of protein sequences in the test dataset with those in the training dataset and assigns the protein function annotation according to the matches with calculated statistical significance.

**FunFams**: Functional Families (FunFams) (Das *et al.*, 2015) is a domain-based method that clusters proteins within the CATH superfamilies into groups that share functions. It can be used to predict the functions of novel protein sequences by scanning all CATH FunFams using HMMER (Eddy, 2009) and transferring all the functions of CATH FunFams with the highest score.

**DeepGO**: DeepGO (Kulmanov, Khan and Hoehndorf, 2018) is one of the proposed protein function annotation methods that apply the architecture of a convolutional neural network followed by a hierarchically structured classifier accounting for dependencies between GO classes. It introduces the constraints of GO relationships by synchronizing the structure of constructed deep multi-layer classifier network with the hierarchical structure of the GO graph and then optimizes the whole hierarchical model in the training process.

**DeepFRI**: DeepFRI (Gligorijević *et al.*, 2021) is a deep-learning-based protein function prediction method that leverages both protein sequence and protein structure information. DeepFRI initially uses a pre-trained Long Short Term Memory (LSTM) (Greff *et al.*, 2016) model to extract protein sequence information, which is then fed into a graph convolutional network of which the structure is organized according to the protein residual-level contact map. The experimental results illustrate a superior prediction performance in protein function annotation.

**TALE+:** TALE+ (Cao and Shen, 2021) is a novel deep learning model ( Transformer-based protein function Annotation through joint sequence–Label Embedding; TALE). TALE+ uses self-attention-based transformers to capture global patterns in protein sequences. For generalizability to unseen or rarely seen functions, TALE+ also embed protein function labels (hierarchical GO terms on directed graphs) together with inputs/features(sequences) in a joint latent space.

**DeepGOZero**: DeepGOZero(Kulmanov and Hoehndorf, 2022) is a machine learning model which improves predictions for functions with no or only a small number of annotations. To achieve this goal, it relied on a model-theoretic approach for learning ontology embedding and combine it with neural networks for protein function prediction. DeepGOZero can exploit formal axioms in the GO to make zero-shot predictions, i.e., predict protein functions even if not a single protein in the training phase was associated with that function.

5. Performance of PFresGO with different ‘hidden_dimension’ values

In this paper, the hypermeter of hidden_dimension in PFresGO was set to 128. We compared the model performance of PFresGO models with hidden_dimension=64 (PFres_dim64) and default hidden_dimension =128 (PFresGO), as well as other two deep-learning-based methods DeepGO and DeepFRI across all GO categories. Our experiment demonstrated that PFresGO (hidden_dimension=128) in general achieved the best performance in terms of AUPRC.

**Supplementary Figure 1**. The precision-recall curves of different methods, including DeepGO, DeepFRI, PFresGO (hidden_dimension =128), and PFres_dim64 (hidden_dimension =64).


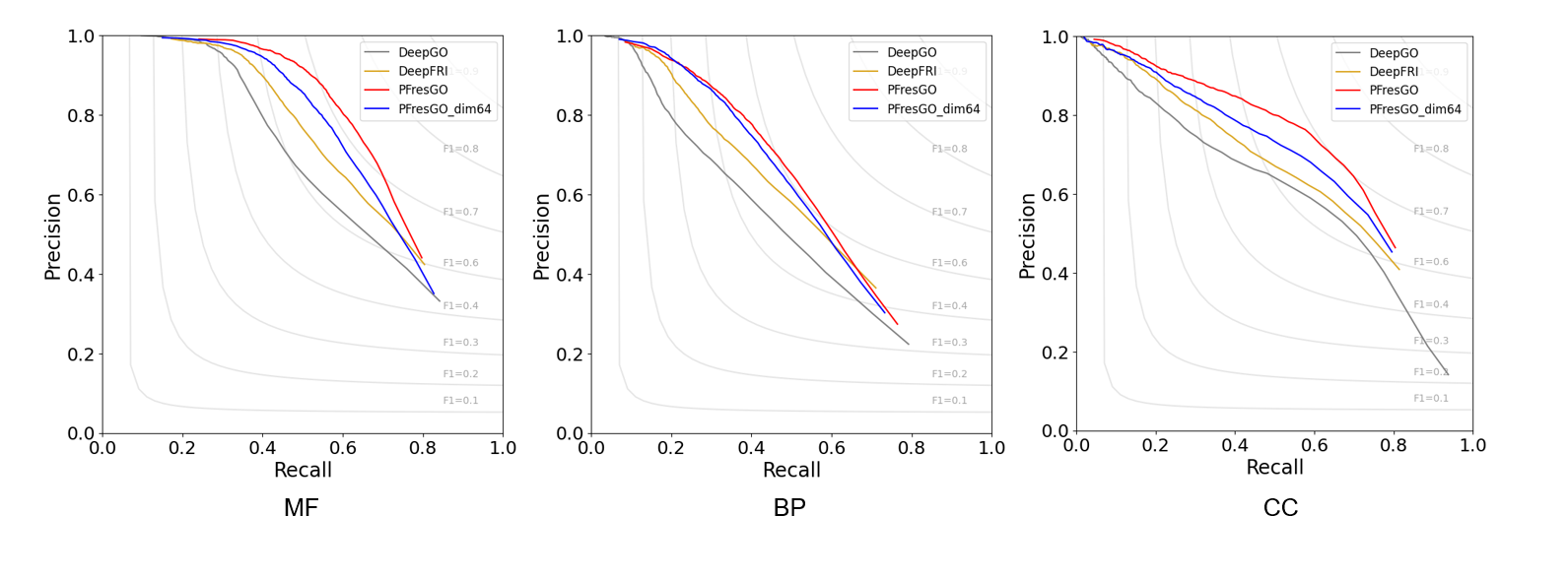


6. Performance of PFresGO with different numbers of layers for the multi-head attention

In this paper, we linked two multi-head attention modules described above for MF and CC protein function annotation. For BP term prediction, only one multi-head attention module was applied considering the memory limitation. We next demonstrated the performance of model with one multi-head attention module (PFresGO_layone) or with two stacked multi-head attention modules (PFresGO) in both MF and CC ontology. We can see form the following figures that model with two stacked multi-head attention modules achieved better performance in term of AUPRC.


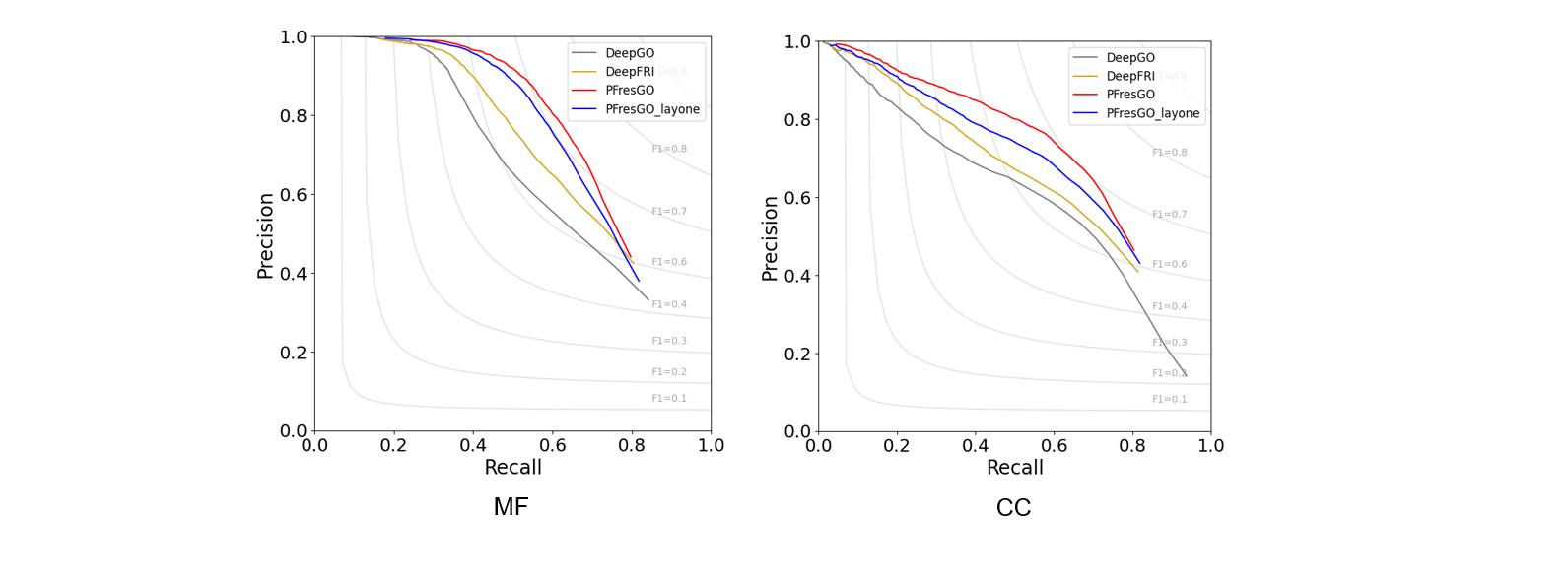


**Supplementary Figure 2**. The precision-recall curves of different methods, including DeepGO, DeepFRI, PFresGO (with two stacked multi-head attention modules), and PFresGO_layone (with one multi-head attention module).

7. Visualization of averaged attention weights

The following figure demonstrates the examples of attention weights of residues in glutathione S-transferase (PDB: 2J9H; Chain A) with function calcium ion binding (left panel; GO:0004364; the red dots correspond to calcium-binding residues annotated in BioLip), and putative cytochrome (PDB: 4RM4; Chain A) for protein function terms ‘heme binding’ (right panel; GO:0020037).


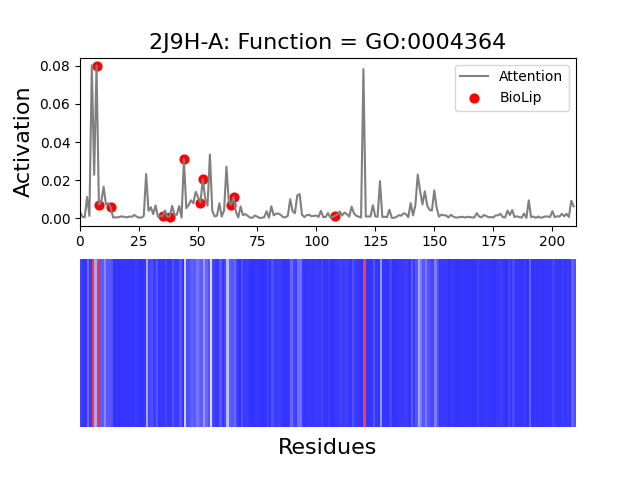

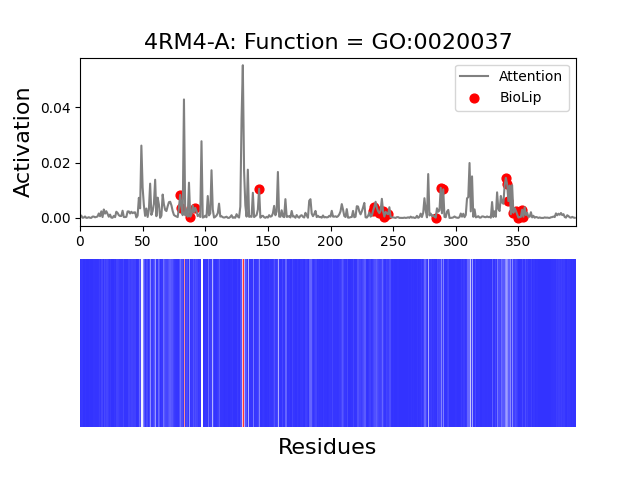


**Supplementary Figure 3.** Visualizing of attention weights of residues

8. Visualization of the percentage of attention on functional sites

We define the following function to compute the percentage of high-confidence attentions that are indicative of protein functional residues to further explore how the attention weights of every head align with known protein functional residues:

$P_{\alpha}\left( f \right)=\frac{\sum_{i=1}^{|X|} f(i)\times\mathbb{A}_{\alpha_{i}>\theta}}{\sum_{i=1}^{|X|} \mathbb{A}_{\alpha_{i}>\theta}}$,

where $f(i)$ is an indicator function that returns 1 if the *i*th residue in the protein sequence X is annotated as a functional site in the BioLip database; otherwise returns 0, $\theta$ ($\theta=0.03here$) represents a threshold used for filtering out the high-confidence residues, and $\mathbb{A}_{\alpha_{i}>\theta}$ indicates the attention weights of the high-confidence residues ($\alpha_{i}>\theta$). The following figures show the proportion of attention weights for protein across two attention layers of PFresGO.


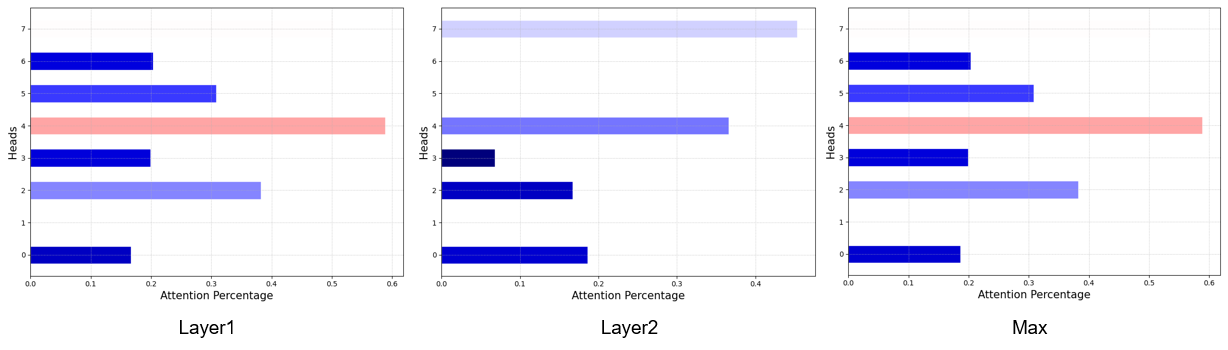


**Supplementary Figure 4.** The percentages of attention of the binding sites of glutathione S-transferase (PDB: 2J9H; Chain A) with function calcium ion binding (GO:0004364). The left, middle, and right bars show the percentages of attentions of every head in attention layer 1, layer 2, and the maximum percentage of each head in layers 1 and 2, respectively.


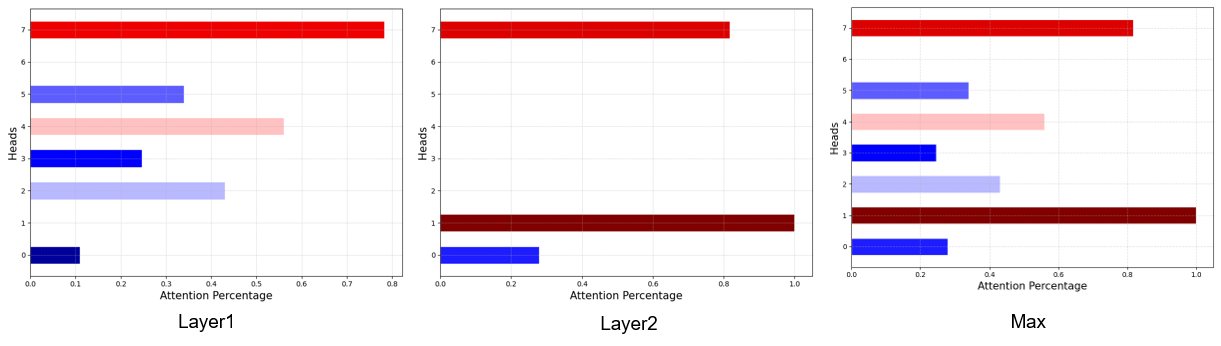


**Supplementary Figure 5.** The percentages of attention of the binding sites of lactose operon repressor (PDB:2PE5, Chain B) with function DNA binding (GO:0003677). The left, middle, and right bars show the percentages of attentions of every head in attention layer 1, layer 2, and the maximum percentage of each head in layers 1 and 2, respectively.


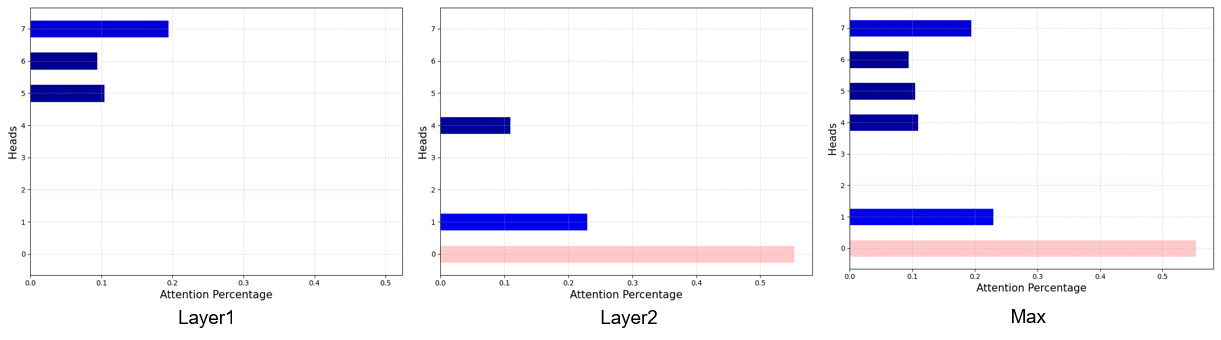


**Supplementary Figure 6.** The percentages of attention of the binding sites of putative cytochrome (PDB: 4RM4; Chain A) with function heme binding (GO:0020037). The left, middle, and right bars show the percentages of attentions of every head in attention layer 1, layer 2, and the maximum percentage of each head in layers 1 and 2, respectively.

9. Performance of PFresGO on dataset divided by ECOD homology level

We used the ECOD (Evolutionary Classification of Domains) (Dustin Schaeffer *et al.*, 2017) homology level to divide our dataset to make different folds sharing no protein domain. Specifically, we used the ECOD classifier (file: “ecod.latest.domains.txt”, version: “20221014”) to rigorously group our dataset protein domains into “homology groups” (the H level). We listed the top 10 groups of H-level classification according to the protein numbers contained in each group in Supplementary Table 2.

**Supplementary Table 2.** Top 10 ECOD hierarchy groups (the H-level) of the PFresGO dataset

| ECOD hierarchy identifier | X-group name | H-group name | Number of proteins |
| --- | --- | --- | --- |
| 2003.1 | Rossmann-like | Rossmann-related | 1953 |
| 2002.1 | TIM beta/alpha-barrel | NO_H_NAME | 1819 |
| 2004.1 | P-loop domains-like | P-loop domains-related | 1399 |
| 11.1 | Immunoglobulin-like beta-sandwich | Immunoglobulin-related | 996 |
| 101.1 | HTH | HTH | 962 |
| 2007.1 | Flavodoxin-like | Class I glutamine amidotransferase-like | 692 |
| 206.1 | NO_X_NAME | Protein kinase/SAICAR synthase/ATP-grasp | 618 |
| 2485.1 | Thioredoxin-like | NO_H_NAME | 597 |
| 1.1 | cradle loop barrel | RIFT-related | 488 |
| 2484.1 | Ribonuclease H-like | NO_H_NAME | 430 |

We split the protein groups with ECOD hierarchy identifiers “2002.1” and “2004.1” out as the test dataset, of which the proteins consisted of around 10% of the whole dataset. Therefore, proteins in the training set shared no ECOD homology domain with the test dataset. We retrained the PFresGO model using this newly divided dataset to analyze protein function annotation performance with enforced criteria that eliminated most if not all evolutionary relationships between our training/validation set and the testing set. The performance values have been listed in Supplementary Table 3.

**Supplementary Table 3.** Performance of PFresGO for protein function prediction on the dataset divided by ECOD (the H-level)

| Approach | GO Category | $F_{max}$ | AUPRC | AUROC | $S_{min}$ |
| --- | --- | --- | --- | --- | --- |
| PFresGO | MF | 0.4342 | 0.1732 | 0.8821 | 2.1580 |
|  | BP | 0.4656 | 0.1049 | 0.9338 | 6.4118 |
|  | CC | 0.2112 | 0.2222 | 0.9742 | 1.5516 |

**References**:

Cao, Y. and Shen, Y. (2021) ‘TALE: Transformer-based protein function Annotation with joint sequence–Label Embedding’, *Bioinformatics*, 37(18), pp. 2825–2833. doi: 10.1093/bioinformatics/btab198.

Das, S. *et al.* (2015) ‘Functional classification of CATH superfamilies: a domain-based approach for protein function annotation’, *Bioinformatics*, 31(21), pp. 3460–3467.

Dustin Schaeffer, R. *et al.* (2017) ‘ECOD: New developments in the evolutionary classification of domains’, *Nucleic Acids Research*, 45(D1), pp. D296–D302. doi: 10.1093/nar/gkw1137.

Eddy, S. R. (2009) ‘A new generation of homology search tools based on probabilistic inference’, in *Genome Informatics 2009: Genome Informatics Series Vol. 23*. World Scientific, pp. 205–211.

Gligorijević, V. *et al.* (2021) ‘Structure-based protein function prediction using graph convolutional networks’, *Nature Communications*, 12(1). doi: 10.1038/s41467-021-23303-9.

Greff, K. *et al.* (2016) ‘LSTM: A search space odyssey’, *IEEE transactions on neural networks and learning systems*, 28(10), pp. 2222–2232.

Kulmanov, M., Khan, M. A. and Hoehndorf, R. (2018) ‘DeepGO: predicting protein functions from sequence and interactions using a deep ontology-aware classifier’, *Bioinformatics*, 34(4), pp. 660–668.

Kulmanov, M. and Hoehndorf, R. DeepGOZero: improving protein function prediction from sequence and zero-shot learning based on ontology axioms. *Bioinformatics* 2022;38(Suppl 1):i238-i245.

Ye, J., McGinnis, S. and Madden, T. L. (2006) ‘BLAST: improvements for better sequence analysis’, *Nucleic acids research*, 34(suppl_2), pp. W6–W9.
